# Analysis of basic pentacysteine6 transcription factor involved in abiotic stress response in *Arabidopsis thaliana*

**DOI:** 10.3389/fgene.2023.1097381

**Published:** 2023-04-17

**Authors:** Zhijun Zhang, Tingting Zhang, Lei Ma

**Affiliations:** College of Life Science, Shihezi University, Shihezi City, Xinjiang, China

**Keywords:** arabidopsis, BPC, abiotic stress, transcription factor, enrichment of function

## Abstract

**Background:** Abiotic stress is a significant environmental factor that limits plant growth. Plants have complex and diverse mechanisms for dealing with abiotic stress, and different response mechanisms are interconnected. Our research aims to find key transcription factors that can respond to multiple non -biological stress.

**Methods:** We used gene expression profile data of Arabidopsis in response to abiotic stress, constructed a weighted gene co-expression network, to obtain key modules in the network. The functions and pathways involved in these modules were further explored by Gene Ontology (GO) and Kyoto Encyclopedia of Genes and Genomes (KEGG) enrichment analyses. Through the enrichment analysis of transcription factor, the transcription factor that plays an important regulatory role in the key module. Through gene difference expression analysis and building protein interaction networks, the important role of key transcription factors is verified.

**Result:** In weighted gene co-expression network, identified three gene modules that are primarily associated with cold stress, heat stress, and salt stress. Functional enrichment analysis indicated that the genes in these modules participate in biological processes such as protein binding, stress response, and others. Transcription factor enrichment analysis revealed that the transcription factor Basic Pentacysteine6 (BPC6) plays a crucial regulatory role in these three modules. The expression of the BPC6 gene is dramatically affected under a variety of abiotic stress treatments, according to an analysis of Arabidopsis gene expression data under abiotic stress treatments. Differential expression analysis showed that there were 57 differentially expressed genes in bpc4 bpc6 double mutant Arabidopsis relative to normal Arabidopsis samples, including 14 BPC6 target genes. Protein interaction network analysis indicated that the differentially expressed genes had strong interactions with BPC6 target genes within the key modules.

**Conclusion:** Our findings reveal that the BPC6 transcription factor plays a key regulatory function in Arabidopsis coping with a variety of abiotic stresses, which opens up new ideas and perspectives for us to understand the mechanism of plants coping with abiotic stresses.

## 1 Introduction

Adverse environmental factors, such as abiotic stress, severely limit agricultural production, reduce crop yield and quality, and affect plant growth and development, thereby threatening food security. Extreme temperatures and soil salinity are common extreme environmental conditions in nature, and climate change further complicates these adverse factors (GONG et al., 2020). Therefore, it is crucial to understand the response mechanism of plants to abiotic stress.

*Arabidopsis* is widely used as a model organism for research in plant genetics, developmental biology and molecular biology. And it is an ideal experimental material for exploring the mechanisms of plant response to abiotic stresses (KILIAN et al., 2007). In previous studies, some important *Arabidopsis* genes and metabolic pathways have been shown that they are involved in the process to respond to abiotic stress with *Arabidopsis*. For example, the *C-repeat Binding Transcription Factor3* (*CBF3*) transcription factor plays a key role in the cold response pathway (HE et al., 2008). Cold-inducible RNA helicase Regulator of *CBF* gene expression1 (*RCF1*) regulates cold-responsive genes and enhances the cold tolerance of plants by clipping pre-mRNA (GUAN et al., 2013). Exogenous application of jasmonate significantly enhances *Arabidopsis* freezing tolerance (HU et al., 2013). Humic acid (HA) significantly induces *Heat Shock Protein-encoding* (*HSP*) genes, including *HSP101*, *HSP81.1*, *HSP26.5*, *HSP23.6*, and *HSP17.6A*, which promotes heat tolerance in *Arabidopsis* (CHA et al., 2020). The *Arabidopsis Temperature-Induced Lipocalins1* (*TIL1*) gene (*AT5G58070*) is an important component of thermotolerance (CHI et al., 2009). Sanguinarine affects heat tolerance in *Arabidopsis* by enhancing the expression of heat shock protein genes such as *HSP17*. *6C-CI*, *HSP70*, and *HSP90.1* (HARA and KURITA, 2014). The *Arabidopsis* histone acetyltransferase *General Control Non-Derepressible5* (*GCN5*) is also an important component of *Arabidopsis* thermotolerance (HU et al., 2015). The *Arabidopsis* regulator *RCF2*, expressed by the *C-repeat Binding Factor* (*CBF*) gene, has been shown to be an integrator of hyperthermia signaling and a mechanism of *Heat Stress Transcription Factor* (*HSF*) and *HSP* activation (GUAN et al., 2014). Overexpression of *Arabidopsis Stress-Induced BTB Protein 1* (*SIBP1*) genes increases salt tolerance in transgenic *Arabidopsis* (WAN et al., 2019). MADS-box transcription factor *Agamous-Linke16* (*AGL16*) acts as a negative regulator in stress response in *Arabidopsis*. The absence of *AGL16* makes *Arabidopsis* resistant to salt stress (ZHAO et al., 2021). Different members of the *Phosphoglycerate Dehydrogenase* (*PGHD*) gene family have different effects on salt tolerance in *Arabidopsis*, and the response to salt stress depends on the specific gene (ROSA-TELLEZ et al., 2020). Many genes or pathways are also involved in the response to multiple abiotic stresses in *Arabidopsis*. Overexpression of *Cysteine2/Histidine2* (*C2H2*)-Type Zinc Finger of *Arabidopsis Thaliana*6 (*ATZAT6*) in *Arabidopsis* can increase resistance to pathogen infection, salt, drought and freeze stress (SHI et al., 2014). DNA methylation is also an important mechanism to regulate abiotic stress resistance in plants (OGNEVA et al., 2019).

Despite the fact that these studies have discovered numerous genes and biological processes in response to abiotic stress, most of these studies have focused on the link between a single gene and a single abiotic stress scenario. However, in nature, stress conditions are frequently layered on a range of unfavorable environmental circumstances. Therefore, the biological processes by which plants respond to different abiotic stresses are not completely independent. Responses to various abiotic stresses are both independent and highly interrelated. Plants possess genes that can respond to several different abiotic stressors simultaneously. To improve plant yield and quality and expand agricultural production, research must be conducted on genes that can adapt to multiple abiotic stresses.

In this study, we retrieved expression data from *Arabidopsis* plants that were subjected to abiotic stress treatments to identify genes that respond to these stressors. We then used the Weighted Gene Co-expression Network Analysis (WGCNA) method to identify the gene modules that are mostly related to cold stress, heat stress, and salt stress. We used a comprehensive bioinformatics approach to analyze the molecular function, signaling pathway, and transcription factor enrichment results of the modules. Finally, we identified a transcription factor, *BPC6* (Basic Pentacysteine), that is highly related to these three stresses. The expression data of *BPC6* under abiotic stress showed that it is involved in *Arabidopsis* responding to various abiotic stresses. Our study will improve the understanding of plant abiotic stress response mechanisms and may play an important role in improving plant yield and quality and promoting agricultural production.

## 2 Result

### 2.1 Data pre-processing

**FIGURE 1:**
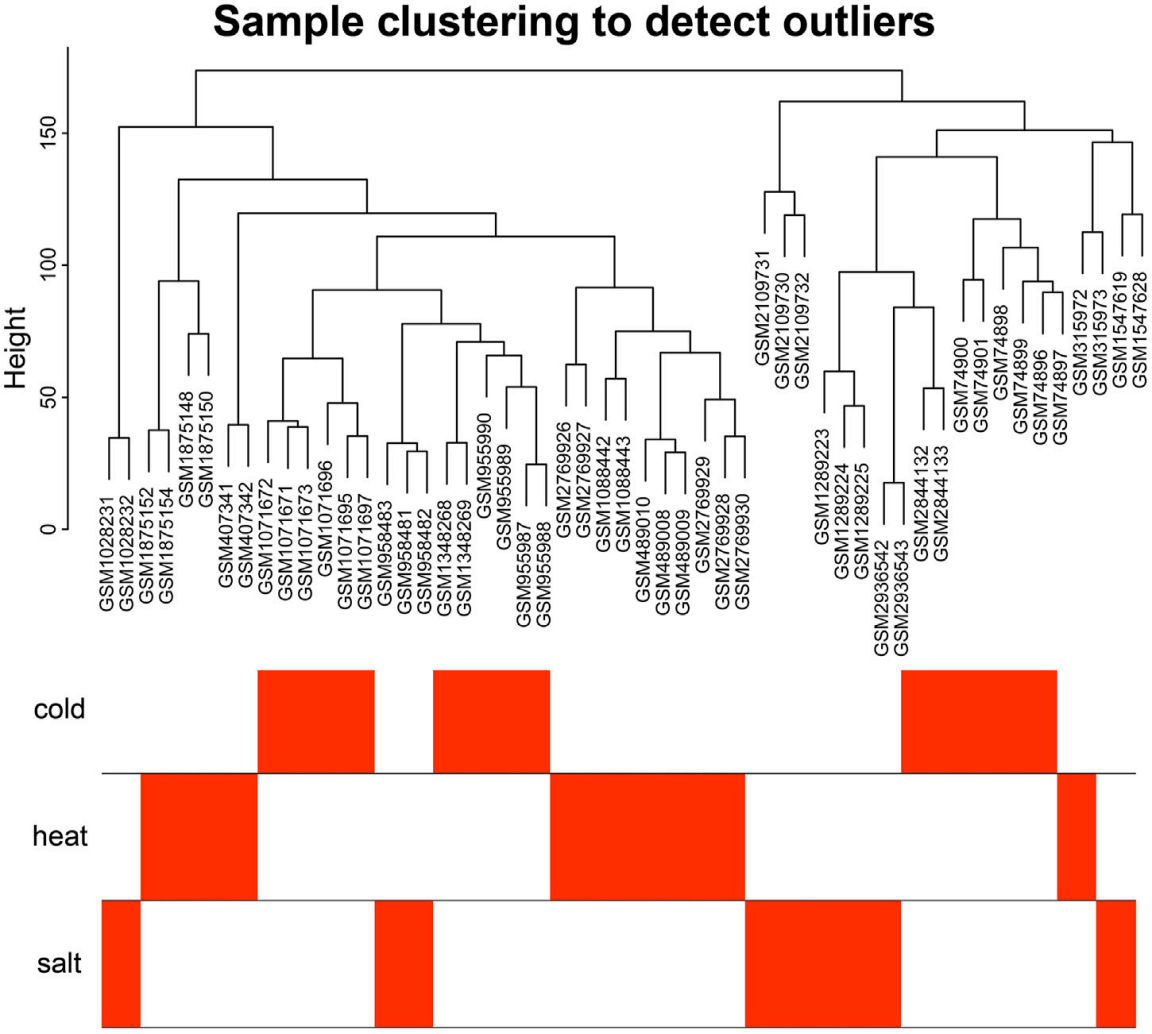
Sample cluster analysis diagram. Sample clustering analysis showed that no samples were outliers.

The 18 gene expression profile data associated with abiotic stress in *Arabidopsis* were preprocessed, and only wild-type *Arabidopsis* samples from all datasets were retained, for a total of 97 samples. All data were normalized. Each dataset contained 20,642 genes. After removing batch effects and putting the 18 datasets together into a new matrix file, all 54 stress-related samples from the new matrix file were selected and control samples were removed. The results of an analysis of Median Absolute Deviation (MAD) are shown in Supplementary Table S1. For building the weighted gene co-expression network, the top 10,000 genes with the most variable expression levels were selected as input genes (mad ≥ 0.390897091). The clustering analysis showed that the 54 *Arabidopsis* samples were close to each other, with no significant outliers (Figure 1), and the overall effect was good.

### 2.2 Weighted gene co-expression network analysis

**FIGURE 2:**
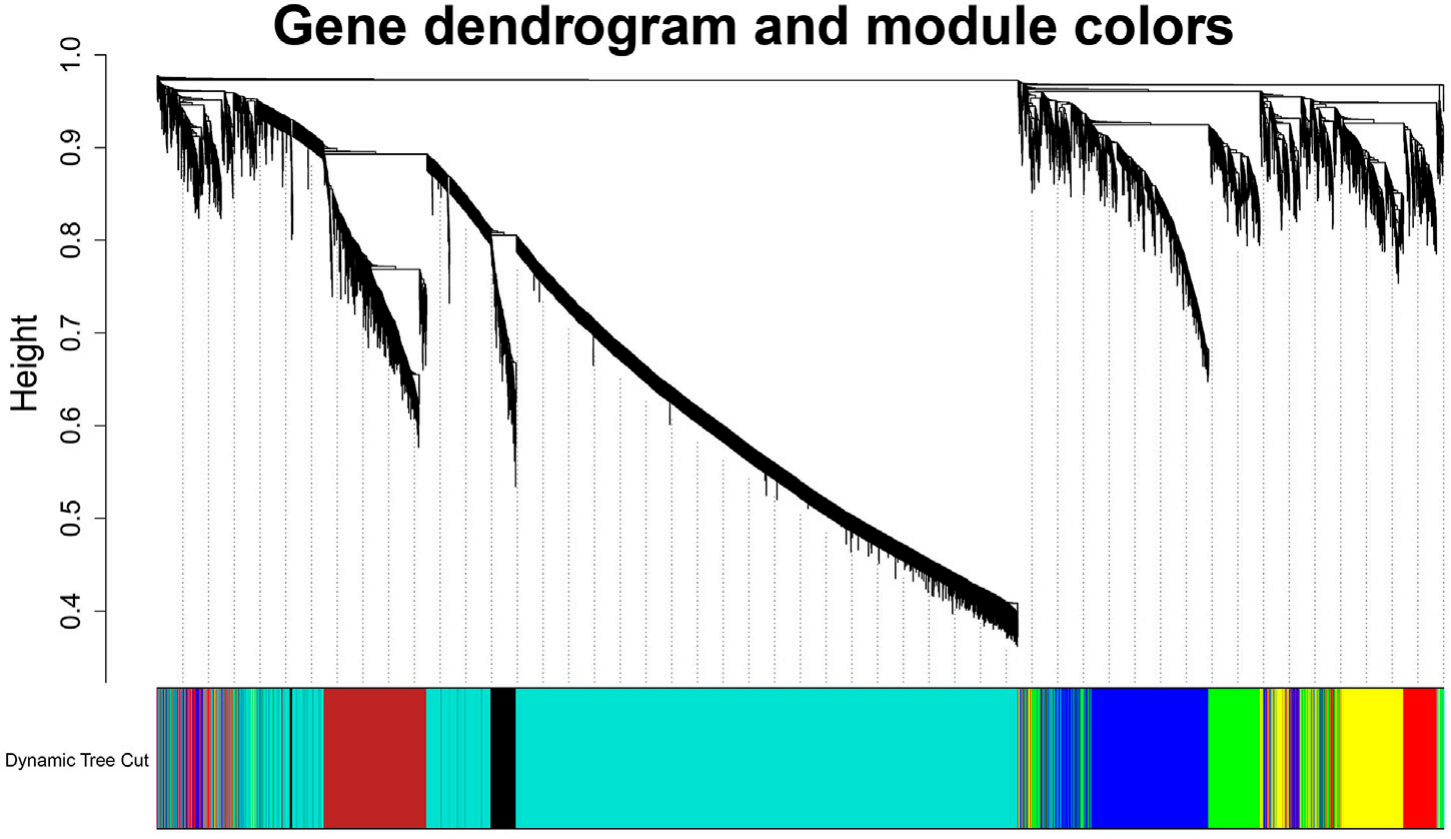
Identification diagram of gene co-expression module. Identification of gene co-expression modules via hierarchical average linkage clustering. The color row underneath the dendrogram shows the module assignment determined by the Dynamic Tree Cut.

To build the scale-free network, we optimized the appropriate network weighting coefficient β. The β was calculated using the “pick Soft Threshold” function of the WGCNA package. When the threshold β was set to 3, the topology analysis showed that the scale-free topology fitting index (R2) was close to 90% (Supplementary Figure S1), indicates that the network was close to being a scale-free network. We established a co-expression network with a soft threshold β of 3. The genes with similar expression patterns were grouped into modules in the network, and a total of seven modules were identified (Figure 2). For visualization, modules were named with colors: Black (258 genes), Blue (1,415 genes), Brown (882 genes), Green (654 genes), Red (571 genes), Turquoise (5,392 genes), and Yellow (827 genes).

**FIGURE 3:**
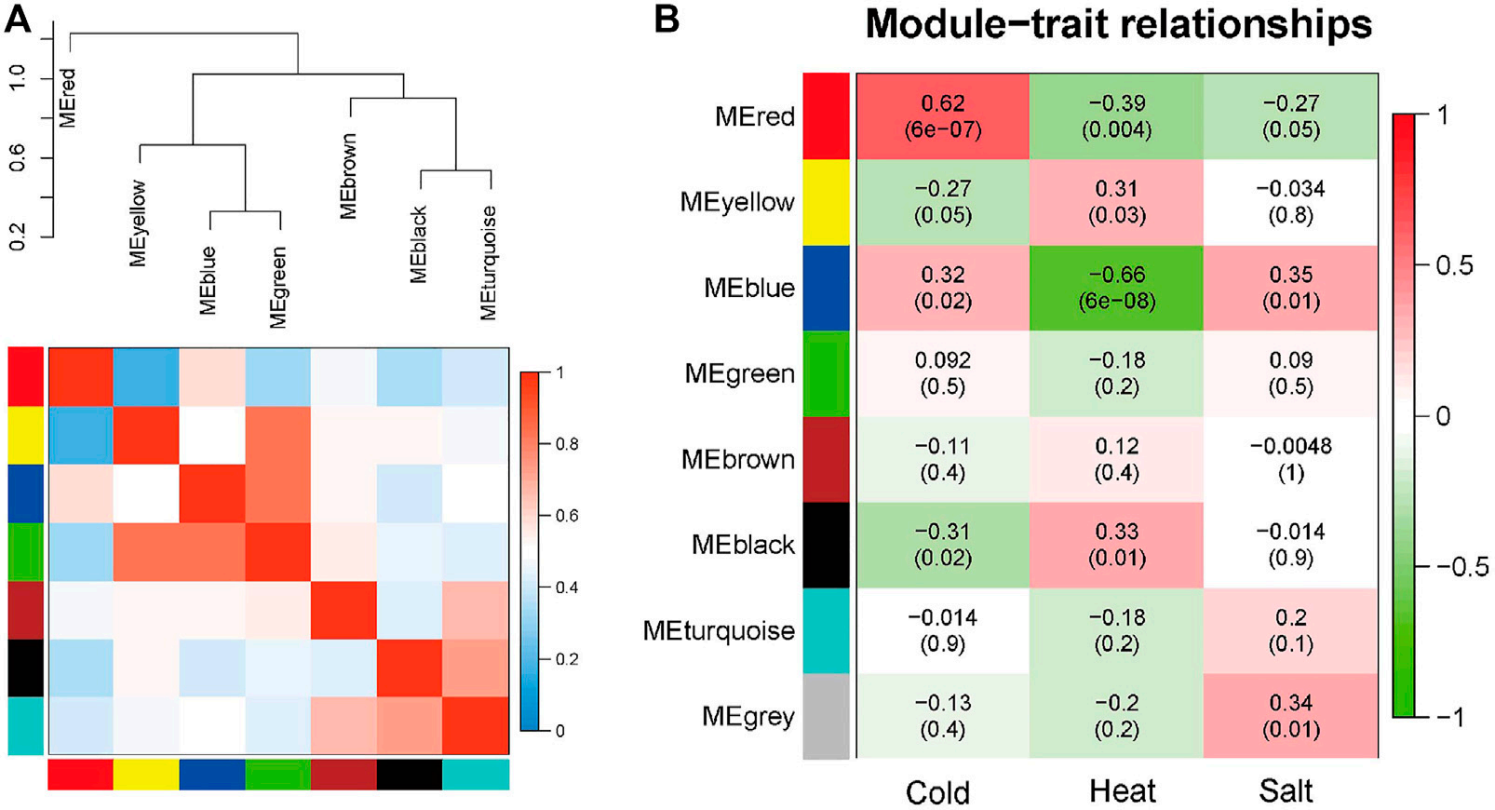
Hierarchical clustering dendrogram of module eigengenes and heatmap plot of the adjacencies in the eigengene network (labeled by their colors). **(A)** In the heatmap, the green color represents low adjacency (negative correlation), while a red represents high adjacency (positive correlation). **(B)** Correlation between sample grouping and gene modules. Each row of the table corresponds to a gene module, and each column corresponds to a group.

The seven modules are primarily divided into three clusters (Figure 3A). Compared to the other modules (Figure 3B), the red module is most closely associated with cold stress, the black module is most closely associated with heat stress, and the blue module is most relevant to salt stress. The results demonstrate that the red (MM = 0.62, *p* = 6e-7), black (MM = 0.33, *p* = 0.01), and blue (MM = 0.35, *p* = 0.01) modules play crucial roles in the *Arabidopsis* response to abiotic stress. Therefore, these three modules are identified as the key modules.

### 2.3 Functional enrichment analysis of key modules

**FIGURE 4:**
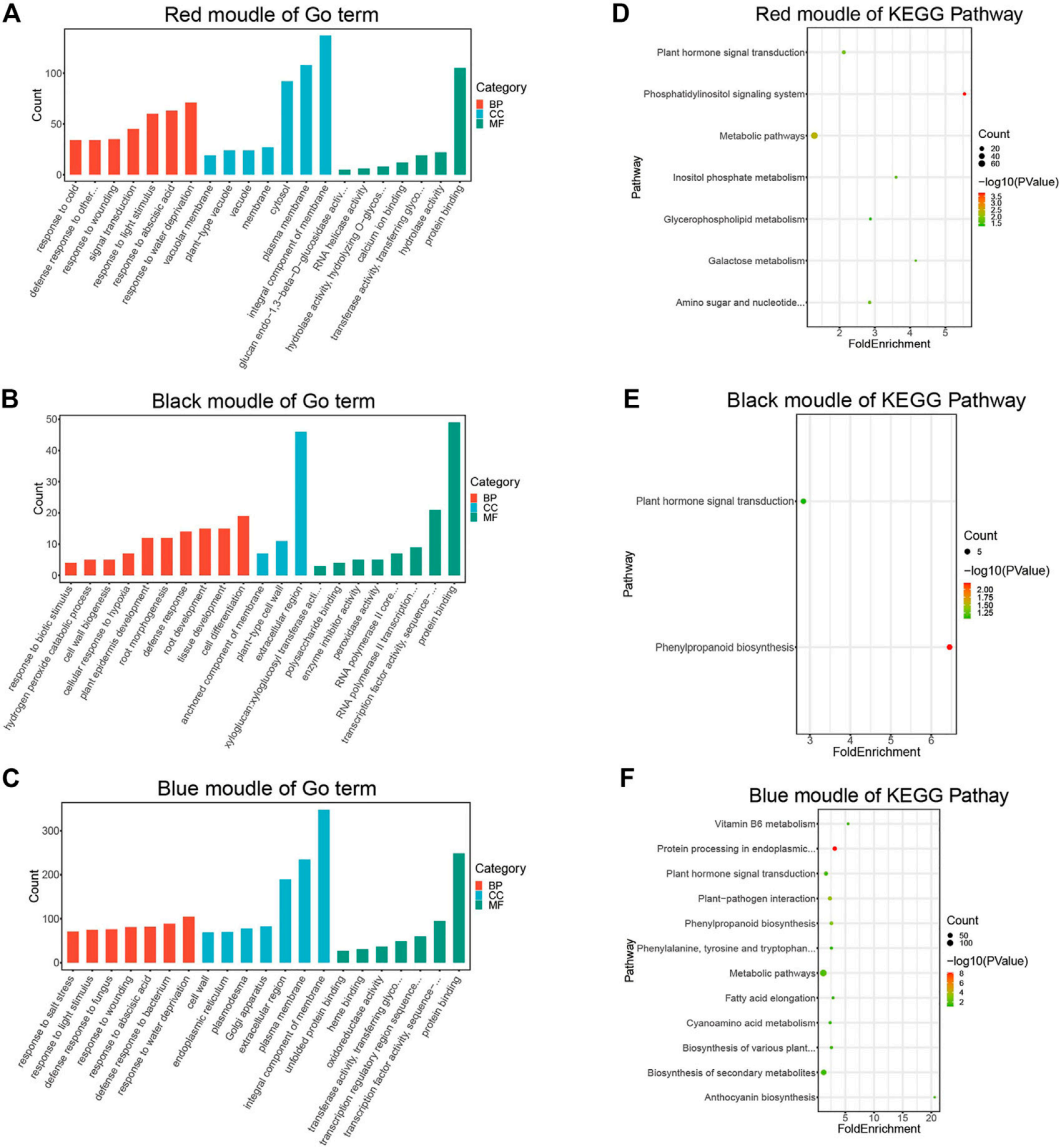
GO and KEGG enrichment analysis diagram. **(A)** GO Enrichment results of genes in the red module. **(B)** GO enrichment results of genes in the black module. **(C)** Enrichment results of genes in the blue module. **(D)** KEGG pathway enrichment results of genes in the red module. **(E)** KEGG pathway enrichment results of genes in the black module. **(F)** KEGG pathway enrichment results of genes in the blue module.

To better understand the biological functions of genes in key modules, the red, black, and blue modules were analyzed for GO function enrichment and KEGG pathway enrichment. The 93 GO terms are significantly enriched in the red module (Figure 4A). For biological processes, genes are mainly concentrated in the response to water shortage, abscisic acid and light stimulation, and signal transduction. For cellular components, genes are mainly enriched in membrane components, the plasma membrane, and cytoplasm. For molecular functions, genes are mainly enriched in protein binding.

The 24 GO terms are significantly enriched in the black module (Figure 4B). For biological processes, genes are mainly enriched in cell differentiation, root development, tissue development, defense responses, and plant epidermis development. For cellular components, genes were mainly enriched in the extracellular region. For molecular functions, genes were mainly enriched in protein transport, transcription factor activity, and sequence-specific DNA binding.

The 178 GO terms are significantly enriched in the blue module (Figure 4C). For biological processes, genes were mainly enriched in response to water shortage, defense response to bacteria, response to injury, and defense response to fungi. For cellular components, genes were mainly enriched in membrane components, the plasma membrane, extracellular region, and Golgi apparatus. For molecular functions, genes were mainly enriched in protein binding, transcription factor activity, sequence-specific DNA binding, and transcriptional regulatory region sequence specific DNA binding.

According to the KEGG pathway analysis, the red module is primarily involved in metabolic pathways (Figure 4D), the black module is mainly associated with phenylpropionic acid biosynthesis (Figure 4E), and the blue module is mainly involved in metabolic pathways and secondary metabolite synthesis (Figure 4F).

### 2.4 Transcription factor enrichment analysis of key modules

**FIGURE 5:**
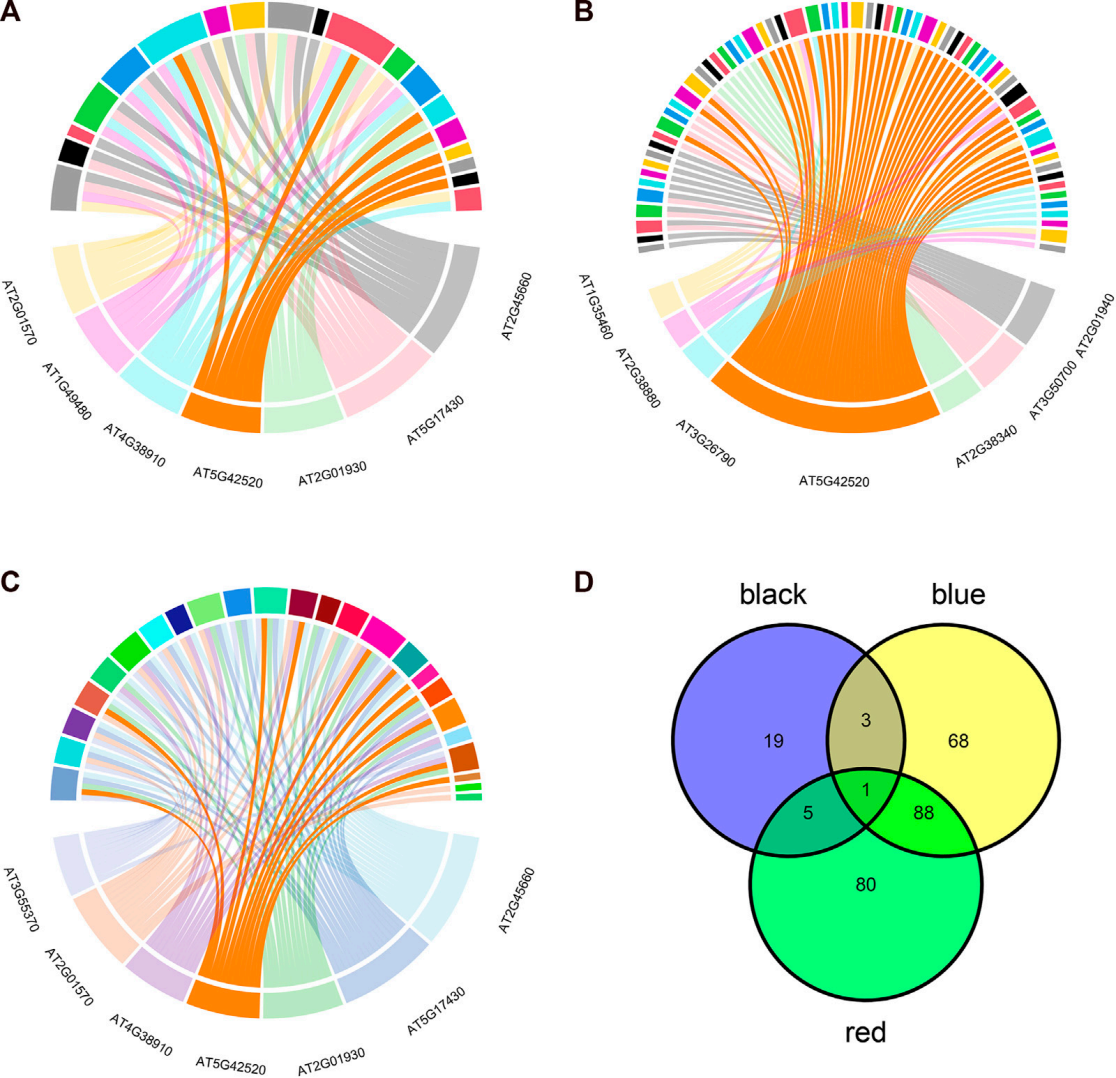
Transcription factor enrichment analysis results. **(A)** Transcription factor enrichment results of genes in the red module. **(B)** Transcription factor enrichment results of genes in the black module. **(C)** Transcription factor enrichment results of genes in the blue module. **(D)** Transcription factor enrichment results are intersected.

To further investigate the common biological mechanisms behind the three key modules responding to abiotic stress, transcription factor enrichment analysis of genes in the three key modules was performed using transcription factor enrichment in PlantTFDB (5.0). Figure 5A shows the enrichment results of the red module, Figure 5B shows the enrichment results of the black module, and Figure 5C shows the enrichment results of the blue module. Supplementary Tables S2–S4 show the regulatory relationship between genes and transcription factors in the red, black, and blue modules, respectively. The intersection of the three enrichment results (Figure 5D) showed that the *BPC6* transcription factor synthesized by the *AT5G42520* gene played a key regulatory role in all three modules simultaneously.

### 2.5 Expression of the *BPC6* gene in *Arabidopsis* under different abiotic stresses

**FIGURE 6:**
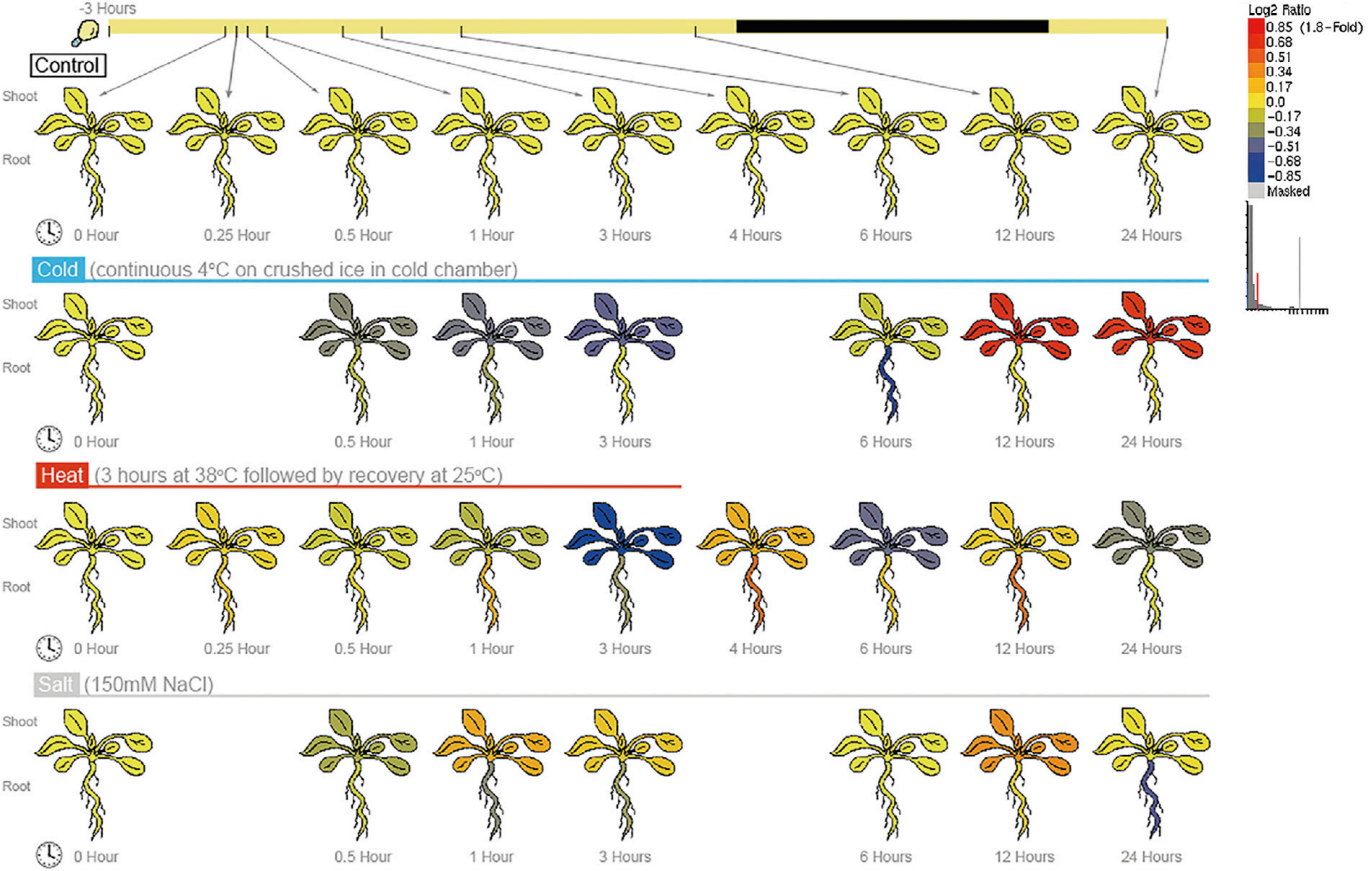
Expression of BPC6 gene under different abiotic stresses.

To test whether the *BPC6* gene plays a key role in *Arabidopsis* responses to multiple abiotic stresses, we analyzed *Arabidopsis* expression profile data from the AtGenExpress project under different abiotic stresses and obtained the expression of the *BPC6* gene under different abiotic stresses (Figure 6). According to the expression profile data. The expression of the *BPC6* gene in *Arabidopsis* decreased significantly during the continuous cold stress period of 4°C. After 6 h of stress, the expression of the *BPC6* gene in plants began to increase significantly, and the change of expression in leaves was more significant. The expression level of the *BPC6* gene in the root continued to decrease initially, but after 6 h, the expression level began to increase and returned to the level before the stress.

During the 38°C/3 h heat stress period, the expression level of the *Arabidopsis* *BPC6* gene slowly decreased. After the stress was stopped, the expression level first increased, then decreased, then increased again, and then decreased once more. At 24 h, the expression level of the *BPC6* gene in the leaves was still low, while the expression level in roots returned to the level before the heat stress.

During the 150 mM/L NaCl salt stress, the expression of the *BPC6* gene in *Arabidopsis* firstly decreased within half an hour of salt stress, then increased within 1 h, then decreased within 6 h, and then increased within 12 h. After 24 h, the expression of the *BPC6* gene in leaves recovered to the pre-stress level, but the expression in roots was significantly lower than that before stress. These results indicated that the *Arabidopsis* *BPC6* gene is involved in the *Arabidopsis* response to various abiotic stresses, which confirms our data analysis results.

### 2.6 Effect of BPC mutant

**FIGURE 7:**
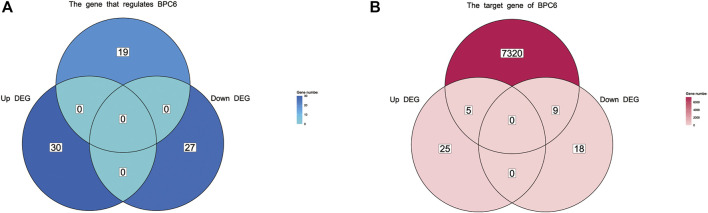
Venn diagram of differentially expressed genes and BPC upstream regulatory genes and downstream target genes. **(A)** Venn diagram of differentially expressed genes and upstream regulatory genes of BPC. **(B)** Venn diagram of differentially expressed genes and target genes regulated by BPC.

After comparing *bpc4 bpc6* double mutant *Arabidopsis* with normal samples, a total of 57 genes were differentially expressed (DEG), with 27 genes being downregulated and 30 genes being upregulated (refer to Supplementary Figure S2, Supplementary Table S7). Out of the 30 upregulated genes, five were identified as *BPC6* target genes according to the plantregmap database, accounting for 20% of all upregulated genes. Similarly, eight of the 27 downregulated genes were *BPC6* target genes, accounting for 29.6% of all downregulated genes (Figure 7B). However, the expression of genes regulating *BPC6* remained unchanged (Figure 7A). These findings suggest that a substantial proportion of the DEGs were target genes regulated by *BPC6*, underscoring the critical role of the *BPC* gene in modulating the expression of these genes.

**FIGURE 8:**
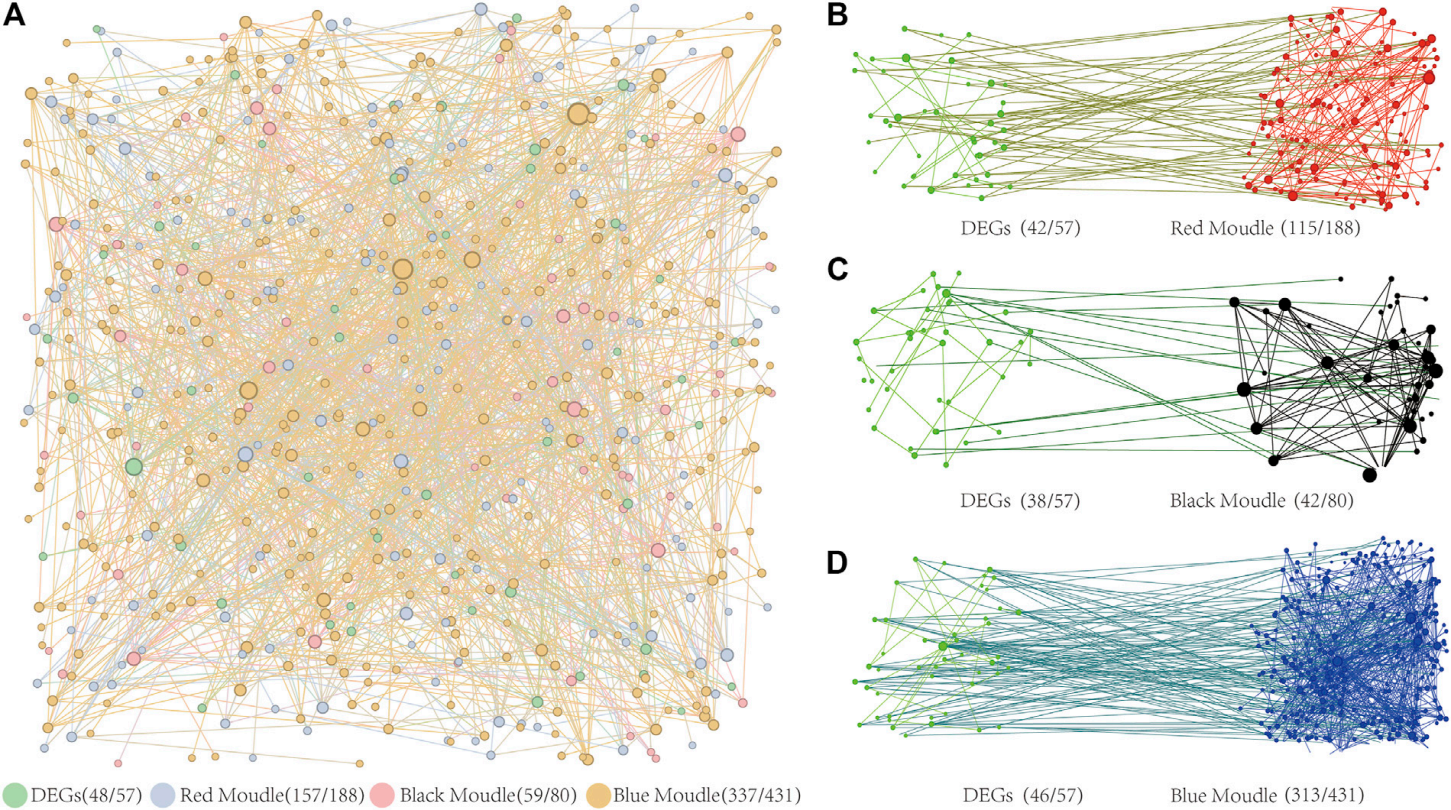
PPI Network Diagram. **(A)** PPI network diagram of three key modules and differentially expressed genes. **(B)** The PPI network diagram of target genes regulated by BPC6 and differentially expressed genes in the red module. **(C)** The PPI network diagram of target genes regulated by BPC6 and differentially expressed genes in the black module. **(D)** The PPI network diagram of target genes regulated by BPC6 and differentially expressed genes in the blue module.

To ensure the accuracy of our findings, we analyzed the Protein-Protein Interaction (PPI) network from both a global and regional perspective. The results of the PPI network analysis of DEGs and target genes controlled by *BPC6* in key modules revealed a significant overall interaction link between these genes (Figure 8A). The PPI network has 48 DEGs, accounting for 84% of the total differential genes. The PPI network contains 157 of the 188 *BPC6* target genes in the red module, accounting for 84% of the total. The PPI network contains 59 of the 80 *BPC6* target genes in the black module, accounting for 74% of the total. The PPI network contains 337 of the 431 *BPC6* target genes in the blue module, accounting for 78% of the total.

PPI network analysis was performed on DEGs and the different key modules. The PPI network analysis results of the red module and 57 DEGs showed that 115 (64%) of the 180 target genes had obvious interactions with 42 (74%) DEGs (Figure 8B). The PPI network analysis results of the black module and 57 DEGs showed that there were obvious interactions between 42 (53%) of the 80 target genes and 38 (67%) DEGs (Figure 8C). The PPI network analysis results of the blue module and 57 DEGs showed that 313 (73%) of the 431 target genes had obvious interactions with 46 (81%) DEGs (Figure 8D). For different key modules, most of the target genes were regulated by *BPC6* and most of the DEGs had obvious interactions.

## 3 Discussion

The ever-increasing global population and the hard-to-increase arable land have aggravated the negative impact on human survival. The best solution to this problem is to increase crop yield per unit area. However, abiotic stress has a strong negative impact on plant growth and crop yield. Abiotic stress factors such as extreme temperatures and soil salinization seriously affect crop production every year, making it significant to improve plant tolerance to abiotic stress. As a model plant, *Arabidopsis* exhibits strong adaptability to environmental stress and is widely used to study various abiotic stress response mechanisms. Most of the previous work has focused on studying *Arabidopsis* response mechanisms to a single stress. However, many extreme climatic conditions occur simultaneously in nature, and the mechanisms by which plants responding to different stresses are not independent of each other. At present, the shared response mechanisms of plants to cope with multiple abiotic stresses are unclear. The *BPC6* transcription factor is an important regulatory transcription factor, and studying its mechanism of participation in coping with abiotic stress is significant.

In this study, we constructed a weighted gene co-expression network using *Arabidopsis* gene expression data and identified three modules that were most associated with cold, heat, and salt stress. The GO enrichment analysis showed that the blue module was mainly involved in the response to water shortage, and had a superior response to bacteria and fungi. The red module was mainly involved in the response to water shortage, abscisic acid, and so on. The black module was mainly involved in cell differentiation, plant development, protein transport, and transcription factor activity. The KEGG pathway enrichment analysis showed that the blue and red modules were mainly involved in the metabolic pathway, while the black module was mainly involved in the phenyl-propionic acid synthesis pathway. The three modules were then enriched for transcription factors, and the results showed that most of the genes in the three modules were simultaneously regulated by the *BPC6* transcription factor. The expression of the *BPC6* gene in *Arabidopsis* was analyzed under different abiotic stresses, and the results showed that the expression of the *BPC6* gene changed significantly under different abiotic stresses.

The DEGs between the *bpc4 bpc6* double mutant *Arabidopsis* samples and normal samples were analyzed. The differential expression analysis shows that compared with normal samples, the *bpc4 bpc6* double mutant *Arabidopsis* must have a total of 57 DEGs. Sequence analysis showed that the *BPC* gene family has a total of 7 genes in *Arabidopsis*, which are divided into three classes: class I proteins *BPC1* (*AT2G01930*), *BPC2* (*AT1G14685*) and *BPC3* (*AT1G68120*); class II proteins *BPC4* (*AT2G21240*), *BPC5* (*AT4G38910*) and *BPC6* (*AT5G42520*); and class III protein *BPC7* (*AT2G35550*). They are all ubiquitously expressed transcriptional activators and repressors, except for *BPC5*, which is considered a pseudogene (MEISTER et al., 2004). There is functional overlap between different classes. Single gene mutations do not produce visible phenotypic effects, and severe morphological phenotypes occur only in higher-order mutants between class I and class II members. Therefore, to study the function of the *BPC6* gene through gene mutant, it is necessary to knock out all *BPC* genes that are similar to the *BPC6* gene function. The control group samples and gene mutant samples used in sequencing are cultivated in normal environments without coercion. Therefore, the only reason for generating differential genes is the mutant of the *BPC4* and *BPC6* genes. The number of different genes is very small. It may be due to the overlapping function of other unintended *BPC* genes with *BPC6*, which caused the physiological biochemical activity of gene knocking *Arabidopsis* not be significantly affected. There are 13 genes among the DEGs that are direct or indirect targets of the *BPC6* transcription factor, accounting for 23% of the total number of different genes. The PPI network analysis of the *BPC6* target genes and DEGs in the key modules can be seen that most of the differences can have a strong interaction with most of the differential genes in the key module in the key module. This proves that the analysis results of the weighted gene co-expression network.

Cytokinin plays an important role in plant growth and development and also participates in the response process of plants to non-biological stress. Research indicates that cytokinins can regulate ion channels, antioxidant enzyme activity, protect chlorophyll and cell membrane stability, and modulate the balance of hormones in plants. The promotes the growth and differentiation of roots, thus increasing plant adaptability to abiotic stress (Sabagh et al., 2021). In addition, cytokinins can also regulate plant abiotic stress responses by interacting with other signaling molecules such as ABA, SA, and ROS (GUPTA and HUANG, 2014; GAO et al., 2019). The type-B *Arabidopsis* response regulator (ARR) transcription factors have emerged as primary targets of cytokinin signaling and are required for essentially all cytokinin-mediated changes in gene expression. By cooperating with other transcription factors, ARR can affect the process and effect of cytokinin in plants (ARGUESO et al., 2010). *BPC* transcription factors are a potential set of coregulators regulating cytokinin responses. Disruption of multiple *BPC* genes in *Arabidopsis* thaliana reduces its sensitivity to cytokinin. Further, a significant number of *BPC6* regulated genes are also direct targets of the type-B ARRs (SHANKS et al., 2018). Therefore, cytokinin is likely to be a key substance involved in *Arabidopsis*’s response to abiotic stress by the *BPC6* transcription factor.

The *BPC* transcription factor family plays a crucial role in regulating gene expression in plants. These proteins are located in the nucleus and regulates the transcription process by specifically binding to the GA dinucleotide repeat sequence of the gene. *BPC* proteins were first discovered in barley in 2003 (SANTI et al., 2003), and subsequently in *Arabidopsis* in 2004 (MEISTER et al., 2004). *BPC* genes have a broad expression pattern in *Arabidopsis*, more than 3,000 *Arabidopsis* genes contain at least one GA-rich segment in their regulatory region. BPC transcription factors are essential for normal plant growth and development. The *Arabidopsis BPC1* transcription factor has been shown to bind to a GA-rich consensus sequence in the Seedstick (*STK*) promoter *in vitro*, and this binding induces conformational changes. Vivo *BPCs* also bind to the consensus boxes, and when these were mutated, expression from the *STK* promoter was derepressed, resulting in ectopic expression in the inflorescence. GA consensus sequences in the *STK* promoter to which *BPCs* bind are essential for the recruitment of the corepressor complex to this promoter (SIMONINI et al., 2012). *Shootmeristemless* (*STM*) and *Brevipedicellus*/*Knat1* (*BP*) genes are both direct targets of *BPC*s, and *BPC* transcription factors also play an important role in the fine regulation of cytokinin content in meristem (SIMONINI and KATER, 2014). *BPC6* can interact with two *Arabidopsis Polycomb-Repressive Complexes* (*PRC1.PRC2*) to affect the expression of a large number of genes (HECKER et al., 2015). *BPCs* can bind to the promoter of transcription factors *Abscisic Acid Insensitive4* (*AAI4*), inhibit the expression of *ABI4* in roots, and promote lateral root (LR) development in *Arabidopsis* (MU et al., 2017). *BPCs* also significantly affect the function of cytokinins in *Arabidopsis*, and disruption of multiple *BPCs* in *Arabidopsis* results in reduced sensitivity to cytokinins (SHANKS et al., 2018). *BPCs* may also promote *Arabidopsis* ovule and seed development by limiting the transcription of *Fusca3* (*FUS3*) (ROSCOE et al., 2019; CARELLA, 2020). Class I *BPC* works by directly binding to the GA/CT cis-element in *FUS3* and limiting its expression (Wu et al., 2020).

Previous studies have demonstrated that the *BPC* transcription factor family plays an important role in regulating plant growth and development. However, from the perspective of abiotic stress, our study expounds a brand-new research result, that is, the *BPC6* transcription factor is involved in the process of plants responding to various abiotic stresses. Compared with previous studies, our advantages lie in the large sample size, abundant data, novel research angles, and diverse research methods. We illustrate new findings with existing data.

Base on further discussion of the results of this study, the following points can be paid attention to: First of all, the specificity and regulatory mechanism of *BPC6* transcription factor in various abiotic stresses can be further explored. Additionally, the interactions and regulatory networks between *BPC6* and other regulators can be studied to gain a deeper understanding of its role in plant abiotic stress responses.

Second, this study found that the blue and red modules were mainly involved in the metabolic pathway, and the black module was mainly involved in the phenylpropionic acid synthesis pathway. This suggests that metabolic and synthetic pathways have important roles in plant responses to abiotic stresses. Therefore, future studies can further focus on the key genes and regulatory mechanisms in these pathways to better understand the physiological and metabolic regulation of plants under abiotic stress.

In addition, the results of this study indicated that different modules have different response characteristics to abiotic stresses. For example, blue modules are mainly involved in the response to water deprivation, while red modules are mainly involved in the response to abscisic acid. This suggests that plants may require different adaptive mechanisms for optimal growth and survival in response to different abiotic stresses. Therefore, future studies could delve deeper into these adaptive mechanisms and response traits to guide plant breeding and planting practices to improve plant adaptability and stress resistance.

Finally, the results of this study demonstrate that key genes and regulatory mechanisms in plants under abiotic stress can be effectively identified using the WGCNA approach. Therefore, future research can apply the WGCNA method to more plant species and different types of abiotic stress to establish a more comprehensive and accurate plant abiotic stress response network, and provide a more scientific basis for plant breeding and cultivation. In addition, by combining other bioinformatics methods, such as gene expression profiling and functional annotation, deeper information and mechanisms of the abiotic stress response network can be further explored. At the same time, since *Arabidopsis* is a model organism, our research results can also guide the study of other plants, which is of great significance for agricultural production and food security.

## 4 Conclusion

The *BPC* transcription factor family is very important in plants and can regulate various plant growth and development processes. From the perspective of abiotic stress, this study explored the role of the *BPC6* transcription factor in *Arabidopsis* response to abiotic stress. It confirmed that *Arabidopsis BPC6* transcription factor can participate in coping with various abiotic stresses by regulating the expression of many genes. Analysis of *Arabidopsis* gene expression data validated this result. This study proves that the biological processes of *Arabidopsis* in response to different abiotic stresses are not isolated, but have commonality at the level of transcription factors. This work provides new ideas and perspectives for the study of plant responses to abiotic stress.

## 5 Materials and methods

### 5.1 Data acquisition

In order to study the mechanism of *Arabidopsis* response to abiotic stress, we searched the GEO (https://www.ncbi.nlm.nih.gov/geo/) database using “*Arabidopsis*” as the keyword. To query gene expression profiles associated with abiotic stress in *Arabidopsis*, we downloaded 18 groups of gene expression profiles related to abiotic stress. These included 6 groups related to salt stress, 6 groups related to heat stress, and 6 groups related to cold stress. We only retained wild-type *Arabidopsis* expression data in the gene expression profiles, resulting in a total of 97 samples. The detailed information of all gene expression profiles is shown in Supplementary Table S8. We also searched the GEO database using “*Arabidopsis*” and “*BPC*” as keywords and obtained gene expression data of *Arabidopsis* thaliana with *BPC* gene mutant (GSE68437). This data set contains eight samples, of which two *bpc4 bpc6* double mutant *Arabidopsis* samples and two control samples were retained. All data used whole plants as material to be sequenced.

### 5.2 Data pre-processing

Gene expression profiles were downloaded in TXT format from the GEO database. The R software package was used to process the matrix files and filter out low-quality data. The probe ID was converted to a gene symbol, invalid expression data were deleted, and the expression data of duplicate gene symbols were averaged. The expression profiles without log2 transformation were log2 transformed using R language. We used the combat package to remove batch effects from all expression profiles, and merged them into a matrix file. The expression data from all stress-treated *Arabidopsis* samples in the matrix file were merged into a new matrix file. Subsequently, we performed WGCNA using the new matrix files containing only stress-treated *Arabidopsis* samples. The GSE68437 dataset was used for gene differential expression analysis.

### 5.3 Weighted gene co-expression network analysis

In statistics, the median absolute deviation (MAD) is a robust measure of sample bias on univariate numerical data. At the same time, it can also represent the population parameters estimated by the MAD of the sample. We used the MAD algorithm to select the expression data of the top 10,000 genes as input data for WGCNA.

WGCNA is regarded as a methodology to reconstruct a free-scale gene co-expression network and concurrently identify modules consisting of highly correlated genes to appraise connectivity between external clinical traits and the module. Eigengene is used for summarizing relationships among internal gene membership. In this study, we applied the one-step network construction and module detection function of the WGCNA package (https://horvath.genetics.ucla.edu/html/Co-expressionNetwork/Rpackages/WGCNA/Tutorials/) in R to handle the analysis of the expression profiles of *Arabidopsis*, which contained 20 cold-treated samples, 18 heat-treated samples, and 15 NaCl-treated samples. We correlated gene clusters with each other and external sample features. The weighted adjacency matrix was calculated to represent the connection strength of each pair of genes. According to the scale-free topology network, the soft thresholding power was set to 4. Then, a hierarchical clustering dendrogram composed of rich branches was established. The dynamic tree-cutting method was used to complete module identification, the minimum size of the gene dendrogram is 25, and the grouping information of samples is made by setting the value of 1 under stress and 0 under no stress as the grouping standard. Finally, modules were associated with groups using module-group associations based on Module Membership (MM) and Gene Salience (GS).

### 5.4 Identification of key modules

We evaluate the relationship between module and sample grouping by using the correlation between module eigengenes and sample grouping. When dealing with sample features, statistical significance measures between module feature genes and features can be defined. For example, using correlation values or *p*-values, modules with high feature significance values are considered to be associated with sample grouping.

### 5.5 Functional and pathway enrichment analysis of key modules

Gene Ontology (GO) enrichment and Kyoto Encyclopedia of Genes and Genomes (KEGG) pathway enrichment analyses of genes in key modules were performed using the online DAVID (https://david.ncifcrf.gov/). The gene list of key modules was uploaded to the DAVID database to obtain the GO enrichment and KEGG pathway enrichment results. Results with *p* < 0.05 were considered significant, and the obtained enrichment analysis results were visualized using the ggplot2 package.

### 5.6 Transcription factor enrichment analysis

Transcription factor enrichment analysis of genes in key modules was performed using the online plantTFDB database (http://planttfdb.gao-lab.org/). The gene list of key modules was uploaded to the plantTFDB database, and the enrichment results of transcription factors of key modules were obtained. R language was used for subsequent analysis of transcription factor enrichment results.

### 5.7 Analysis of key gene expression

*Arabidopsis* eFP Browsers (http://bar.utoronto.ca/#GeneExpressionAndProteinTools) from the AtGenExpress project were used to analyze the expression profiles of *Arabidopsis* genes under different abiotic stresses, using the *Arabidopsis* eFP in the BAR database (KILIAN et al., 2007).

### 5.8 Gene expression data validation

To verify our data analysis results, we used the plantregmap database (http://plantregmap.gao-lab.org/) to obtain all target genes regulated by *BPC6* and all genes that regulate *BPC6* in *Arabidopsis*. We also used the limma package (https://bioconductor.org/packages/release/bioc/html/limma.html) to analyze the gene differential expression of the which two *bpc4 bpc6* double mutant *Arabidopsis* samples and normal samples in the GSE68437 dataset (|log_2_^FC^| > 2, adj.*p* < 0.05). Additionally, we utilized the STRING (https://string-db.org/) to perform PPI networks analysis on DEGs and key genes within the three modules, and then used Gephi V0.10.1 to visualize the PPI network. Finally, we took the intersection of *BPC6*-related genes and DEGs to verify our data analysis results.

## Data Availability

The datasets presented in this study can be found in online repositories. The names of the repository/repositories and accession number(s) can be found in the article/Supplementary Material.

## Supplementary material

SUPPLEMENTARY FIGURE S1 Soft threshold analysis result.

SUPPLEMENTARY FIGURE S2 Volcano plot of differentially expressed genes.

SUPPLEMENTARY TABLE S1 Median Absolute Deviation (MAD) analysis results.

SUPPLEMENTARY TABLE S2 Regulatory relationship between genes and transcription factors in red module.

SUPPLEMENTARY TABLE S3 Regulatory relationship between genes and transcription factors in black module.

SUPPLEMENTARY TABLE S4 Regulatory relationship between genes and transcription factors in blue module.

SUPPLEMENTARY TABLE S5 The gene list and regulatory relationship that can regulate BPC6 in Arabidopsis.

SUPPLEMENTARY TABLE S6 The target gene and regulatory relationship of BPC6 in Arabidopsis.

SUPPLEMENTARY TABLE S7 Results of gene differential expression analysis.

SUPPLEMENTARY TABLE S8 Detailed information about GEO data.
