# Supplementary figures and images for: Analysis of basic pentacysteine6 transcription factor involved in abiotic stress response in Arabidopsis thaliana
Source: Front Genet. 2023 Apr 17;14:1097381. doi: 10.3389/fgene.2023.1097381 (PMC10150019; doi:10.3389/fgene.2023.1097381)

Volcano

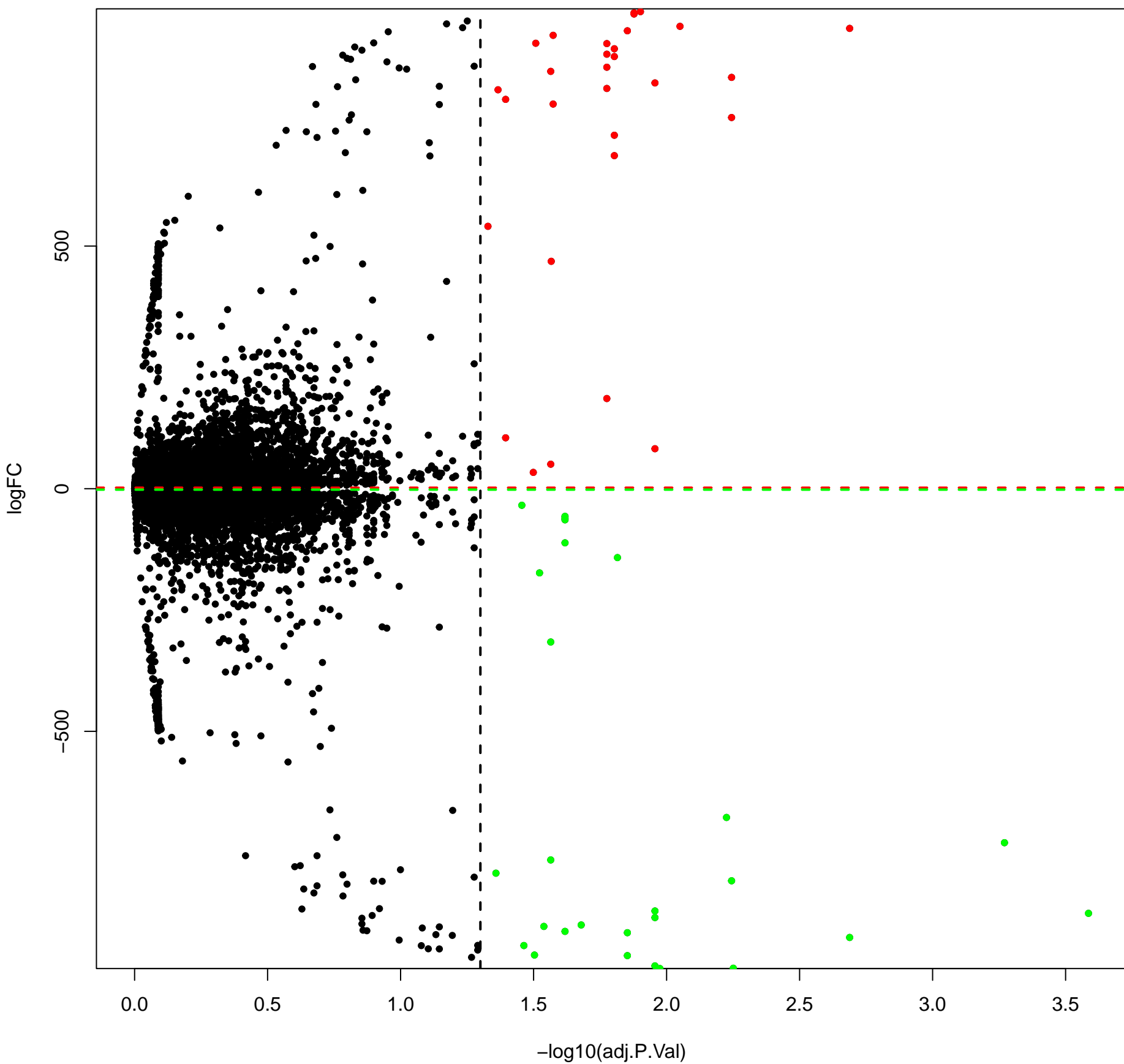

Supplement: Supplementary file 1 [file DataSheet2.PDF]

## Scale independence

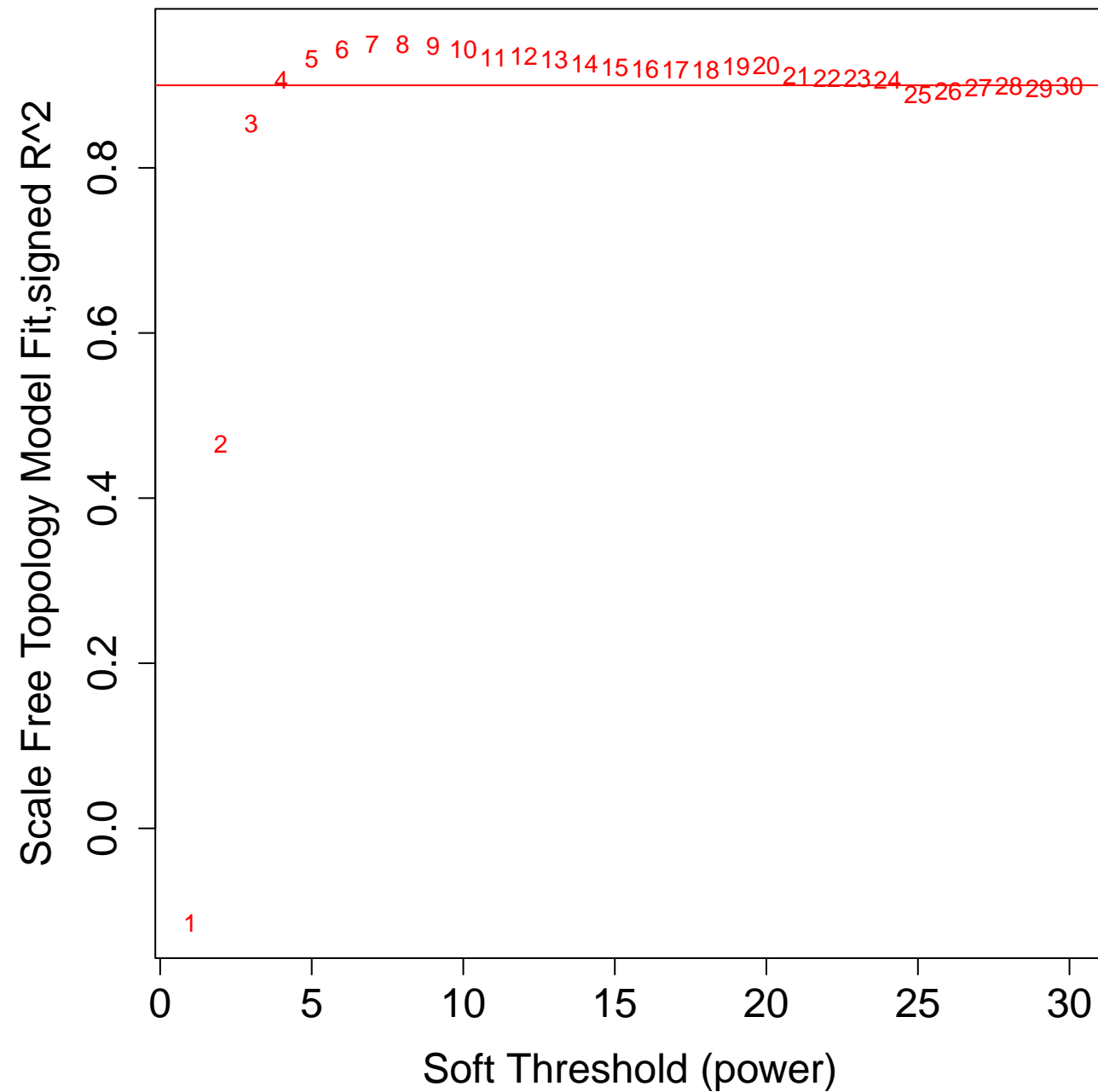

## Mean connectivity

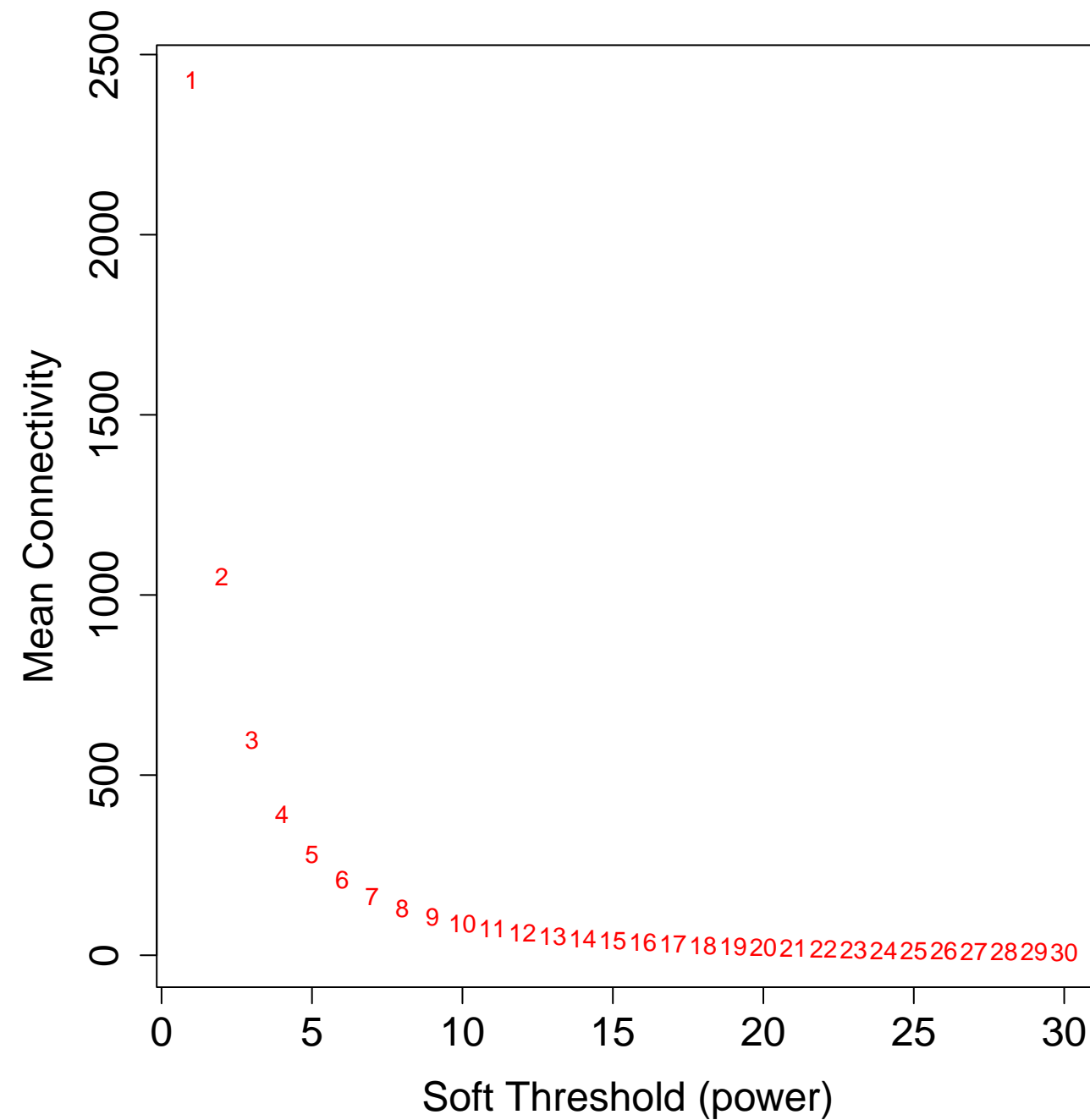

Supplement: Supplementary file 7 [file DataSheet1.PDF]
