# Supplementary material for: Analysis of basic pentacysteine6 transcription factor involved in abiotic stress response in Arabidopsis thaliana
Source: Front Genet. 2023 Apr 17;14:1097381. doi: 10.3389/fgene.2023.1097381 (PMC10150019; doi:10.3389/fgene.2023.1097381)
Supplement: Supplementary file 5 [file Table8.DOCX]

| Dataset | Stress | Platform | Number of samples (Treatment/Control) | Organism |
| --- | --- | --- | --- | --- |
| GSE3326 | Cold | GPL198 | 8(6/2) | Arabidopsis thaliana |
| GSE39090 | Cold | GPL198 | 6(4/2) | Arabidopsis thaliana |
| GSE43818 | Cold | GPL198 | 6(3/3) | Arabidopsis thaliana |
| GSE43819 | Cold | GPL198 | 6(3/3) | Arabidopsis thaliana |
| GSE55907 | Cold | GPL198 | 4(2/2) | Arabidopsis thaliana |
| GSE106635 | Cold | GPL198 | 4(2/2) | Arabidopsis thaliana |
| GSE12619 | Heat | GPL198 | 4(2/2) | Arabidopsis thaliana |
| GSE16222 | Heat | GPL198 | 4(2/2) | Arabidopsis thaliana |
| GSE19603 | Heat | GPL198 | 5(3/2) | Arabidopsis thaliana |
| GSE44655 | Heat | GPL198 | 4(2/2) | Arabidopsis thaliana |
| GSE74929 | Heat | GPL198 | 8(4/4) | Arabidopsis thaliana |
| GSE103398 | Heat | GPL198 | 8(5/3) | Arabidopsis thaliana |
| GSE39236 | Salt | GPL198 | 6(3/3) | Arabidopsis thaliana |
| GSE41963 | Salt | GPL198 | 4(2/2) | Arabidopsis thaliana |
| GSE53308 | Salt | GPL198 | 6(3/3) | Arabidopsis thaliana |
| GSE63372 | Salt | GPL198 | 4(2/2) | Arabidopsis thaliana |
| GSE79997 | Salt | GPL198 | 6(3/3) | Arabidopsis thaliana |
| GSE109283 | Salt | GPL198 | 4(2/2) | Arabidopsis thaliana |
| GSE68437 | gene knockout | GPL198 | 8(2/2) | Arabidopsis thaliana |
